# Overview of Chagas disease in Amazonas: an integrated approach

**DOI:** 10.1590/0037-8682-0581-2025

**Published:** 2026-08-07

**Authors:** Maria das Graças Vale Barbosa Guerra, Flávio Renan Paula da Costa Alcântara, Alba Regina Jorge Brandão, Rômulo Freire de Morais, Jessica Vanina Ortiz, Gabriela Maciel Alencar, Nádelly Karoline Martins Derze, Cássia Camila de Oliveira Araújo, Lara Isabelli Oliveira da Silva, Susan Smith Doria, Lucely Paiva Rodrigues da Silva, Mônica Regina Hosannah da Silva e Silva, Kátia do Nascimento Couceiro, João Marcos Bemfica Barbosa Ferreira, Jorge Augusto de Oliveira Guerra

**Affiliations:** 1 Universidade do Estado do Amazonas (UEA), Programa de Pós-graduação em Medicina Tropical, Manaus, AM, Brasil.; 2 Universidade do Estado do Amazonas (UEA), Programa de Ciências da Saúde da Amazônia, Manaus, AM, Brasil.; 3 Universidade do Estado do Amazonas (UEA), Programa de Pós-graduação em Ciências Aplicadas à Dermatologia, Manaus, AM, Brasil.; 4 Universidade Federal do Amazonas (UFAM), Manaus, AM, Brasil.; 5 Fundação de Medicina Tropical Dr. Heitor Vieira Dourado (FMT-HVD), Manaus, AM, Brasil.

**Keywords:** Trypanosoma cruzi, Amazonas, Chagas disease surveillance

## Abstract

In the Amazon, Chagas disease (CD) transmission is associated with the consumption of contaminated foods -most notably açaí-and constitutes an important public health concern. In Brazil, the state of Amazonas ranks third in acute-phase notifications among states in the region, with 25% of municipalities having already reported acute CD cases, predominantly in outbreaks in rural areas. This study describes the current overview of knowledge on CD in Amazonas, bringing together research findings from the Fundação de Medicina Tropical Doutor Heitor Vieira Dourado (FMT-HVD), developed in partnership with the graduate program in Tropical Medicine at the Universidade do Estado do Amazonas (UEA). The findings show that, in addition to oral transmission, accidental contact with the traditional vector (Triatominae) has been reported. The predominant transmission pattern represents an important shift in the dynamics of the disease and reinforces the need for surveillance strategies focused on controlling foodborne transmission. Among the main challenges are logistical difficulties for post-treatment clinical follow-up, especially in remote areas, and the absence of a structured and specialized network for continuous case monitoring and detection of new outbreaks. Added to this is the lack of systematic CD screening in women of childbearing age during prenatal care, which hinders surveillance of vertical transmission. These issues highlight the urgency of strengthening surveillance and preventing new incidences.

## INTRODUCTION

Chagas disease (CD), previously restricted to rural areas of Latin America, has expanded to other continents via migratory processes. It is estimated that currently more than 7 million people are infected worldwide, making early diagnosis essential^1^. Although *Trypanosoma cruzi* has long been present in the Amazon, from the late 1990s onward, outbreaks of orally transmitted infections - mainly associated with contaminated açaí - have surged, posing a growing public-health concern^2^. 

**FIGURE 1:**
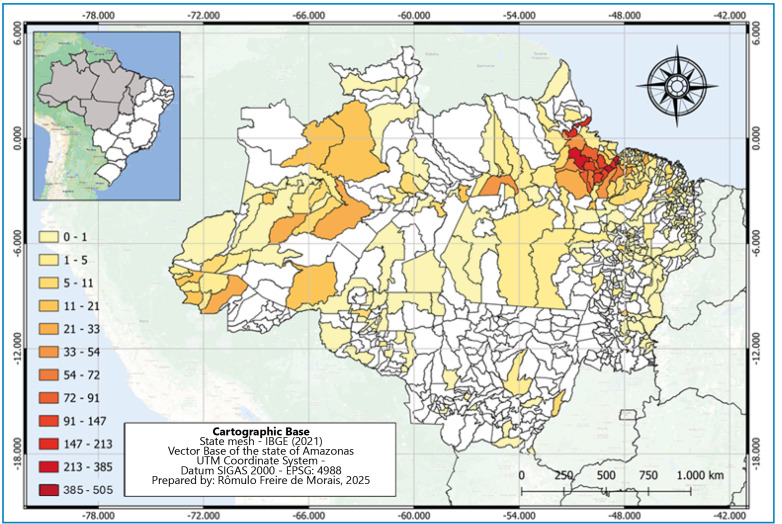
Geographic distribution of acute Chagas disease cases in the states of the Legal Amazon between 2003-2023. Source: SINAN (2025); FMT-HVD (2025).

The Legal Amazon represents about 59% of Brazil and 67% of the Pan-Amazon, which encompasses nine countries, more than 30 million inhabitants and 44% of South America^3^. Established by Law No. 1,806/1953, the Legal Amazon includes 9 states and 772 municipalities, of which 192 (25%) have already reported acute cases of CD (Figure 1). Of these, 80% were in the state of Pará, 7% in Amapá, 6% in Amazonas and the remaining states account for 7% of the cases^2^. In this region, CD exhibits epidemiological characteristics that differ markedly from those observed in Brazil’s traditional endemic regions^4^. 

The biological cycle of *Trypanosoma cruzi* is strictly sylvatic, with no household colonization by vectors, and household vector transmission is accidental. Data from the Notifiable Diseases Information System (SINAN) show that between 2003 and 2022 more than 3,700 acute CD cases were reported, 80% of which were associated with oral transmission through the consumption of contaminated foods, mainly açaí^2^. 

## TEMPORAL EVOLUTION AND CURRENT SITUATION OF CHAGAS DISEASE IN AMAZONAS

### History of Chagas disease in Amazonas

In Amazonas, CD was first documented in 1977, when, using diagnosis via serology, six chronic cases were identified among palm-fiber collectors in Barcelos, in the upper Negro River region^5^. *Leopoldinia piassaba* (Wallace) is a species of palm trees that is endemic to the Negro River basin^6^ and serves as a natural ecotope for a triatomine species, *Rhodnius brethesi* ^7^. In 1979, in this state, the first case of CD in the acute phase was diagnosed in the municipality of São Paulo de Olivença, with suspected vector transmission^8^. In the following years, isolated cases appeared sporadically^4^. In 2004, in the municipality of Tefé, the first orally transmitted outbreak was recorded, associated with the consumption of açaí. On that occasion, nine people were diagnosed. From this event onward, isolated cases^4^ and acute CD outbreaks associated with oral transmission have been recorded^9^ ^,^ ^10^. Currently, the Amazonas state reports the third-highest number of acute Chagas disease cases in the Brazilian Amazon, with rising numbers primarily driven by oral transmission^2^. 

**FIGURE 2:**
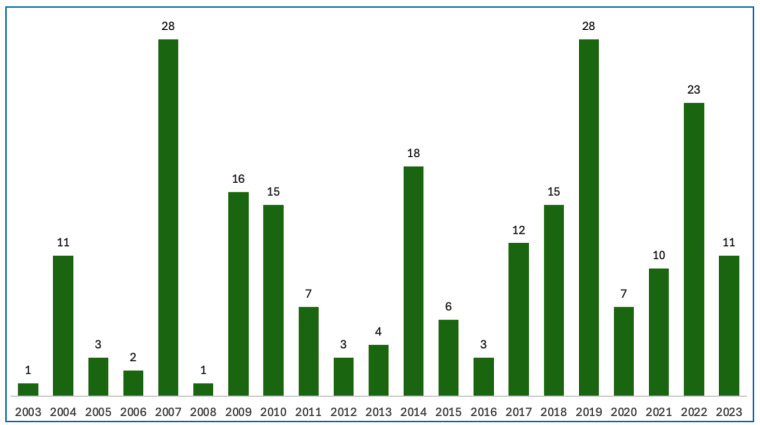
Distribution of acute Chagas disease cases in the state of Amazonas between 2003-2023. **Source:** SINAN (2025) and FMT-HVD (2025).

**FIGURE 3:**
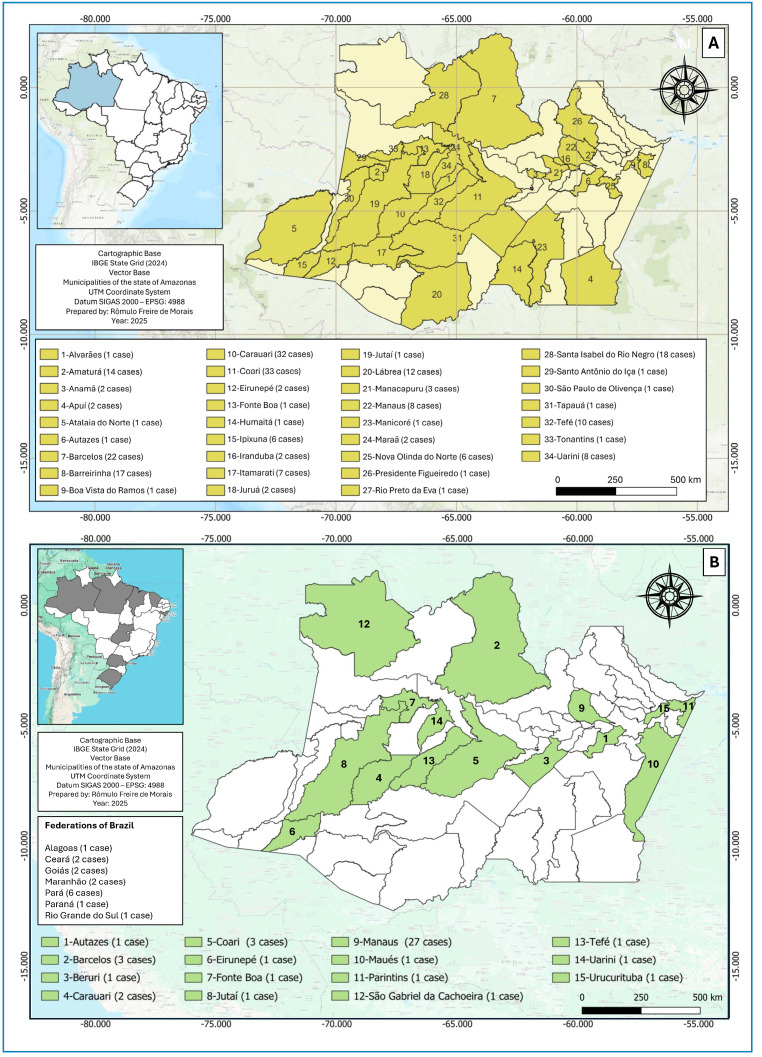
Spatial distribution of acute Chagas disease cases in the state of Amazonas between 2003-2023 **(A)**. Source: SINAN (2025). Spatial distribution of chronic Chagas disease cases in the state of Amazonas between 2009-2024 **(B)**. **Source:** FMT-HVD data

In this state, the Fundação de Medicina Tropical Doutor Heitor Vieira Dourado (FMT-HVD) is the reference center for the care, diagnosis and treatment of CD patients and, together with the Fundação de Vigilância em Saúde do Amazonas Dra. Rosemary Costa Pinto (FVS-RCP) conducts investigations into acute CD outbreaks^4^. According to SINAN^2^ data and the outpatient care service of FMT-HVD^10^, 223 acute CD cases were recorded in the state of Amazonas between 2003 and 2023, and it is observed that the identification of these cases increased significantly from 2004 onwards (Figure 2), the year in which actions to detect *T. cruzi* in the malaria surveillance network were intensified^11^. By 2023, 34 (53%) of the 62 municipalities had already reported the occurrence of acute-phase cases^2^ (Figure 3).

**FIGURE 4:**
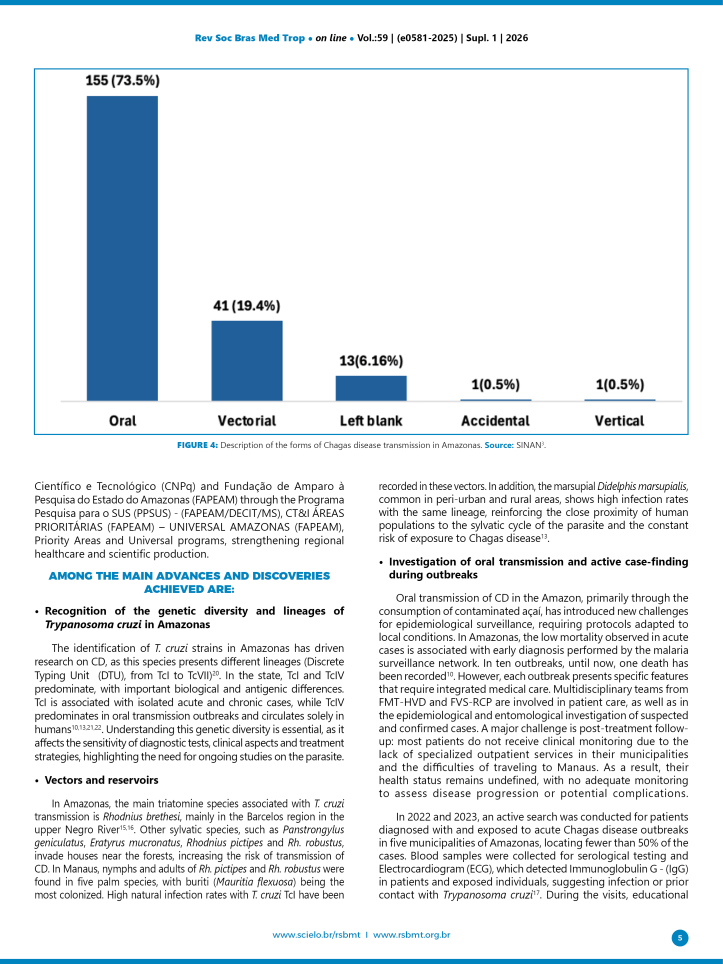
Description of the forms of Chagas disease transmission in Amazonas. **Source:** SINAN^3^.

With regard of chronic Chagas disease the monitoring began in 2007, following the first serological survey conducted in Manaus, Coari, and Tefé^12^. This led to the creation of the CD outpatient clinic at FMT-HVD, which is responsible for the clinical follow-up of diagnosed patients, including treated acute cases, individuals identified through serological surveys, blood donors with reactive serology Fundação Hospitalar de Hematologia e Hemoterapia do Amazonas (FHEMOAM), and cardiology referrals. According to a recent survey, the outpatient clinic registers chronic patients coming from other states and from 15 (24%) of the 62 municipalities of Amazonas, highlighting the regional scope of specialized care (Figure 4).

### Definition of the forms of transmission of Chagas disease in Amazonas

In Amazonas, triatomines have strictly sylvatic habits, but occasionally adult *Rhodnius robustus*, *Rh. pictipes* and *Panstrongylus geniculatus* are found in both urban and rural houses near the forest^13^. These nocturnal insects are attracted to house lights, increasing the risk of accidental vector transmission. It is noteworthy that the natural infection rate of *T. cruzi* in these vectors is high. It is estimated that thousands of new infections occur annually in the Americas; however, most remain undetected by surveillance systems due to their asymptomatic nature^14^, This condition of asymptomatic CD cases is relevant because, even if they do not present symptoms during the acute phase, these individuals can develop chronic complications over time, which underscores the need to understand the true prevalence of infection, including these silent cases, for a more accurate assessment of the disease's impact^15^.

*Vector transmission -* In Amazonas, the suspicion of household vector transmission occurs when a person is diagnosed in isolation, with no other associated case. Vector transmission is confirmed when there is evidence of a *T. cruzi* entry point, such as an inoculation chagoma or Romaña’s sign. However, these signs are rare. Over the past 20 years, at least 27 isolated acute CD cases have been diagnosed and treated at FMT-HVD, originating from 10 (16%) of the municipalities of this state. Among all the patients seen at the outpatient clinic, an inoculation chagoma was observed in two children and Romaña’s sign in two women. 

*Oral transmission -* Oral transmission is currently the form that generates the highest number of acute CD cases. Since 2004, when the first acute disease outbreak was recorded, associated with the consumption of açaí (*Euterpe* sp.) juice contaminated with *T. cruzi* ^10^, this form of transmission has accounted for 73.5% of reported cases, while vector transmission represents about 19.4% of cases^2^ (**Figure 4**). 

### Transmission in the chronic phase of Chagas disease

In chronic CD, it is generally not possible to precisely identify the route of transmission, as individuals discover they are infected without knowing when they were exposed to *T. cruzi*. In these situations, vector-borne transmission is suspected. Silent vector-borne transmission cases pose a significant challenge to the assessment of CD, highlighting the need for more sensitive surveillance strategies to detect these subclinical or asymptomatic cases^15^. In the municipality of Barcelos, Amazonas, there is strong evidence of vector transmission in chronic cases, mainly among piassava fiber (*Leopoldinia piassaba* Wallace) extractivists, a palm species used as a natural ecotope for the triatomine *Rhodnius brethesi*. The so-called piassava workers maintain frequent contact with these vectors, and thus their work directly exposes them to the *T. cruzi* cycle in the region^16^. For this reason, this form of transmission is associated with both acute and chronic CD cases diagnosed in the area, reinforcing the need for continuous vector surveillance in the region^17^.

### Clinical and epidemiological profile of Chagas disease patients

In general, among patients seen at the CD outpatient clinic, there are people of either sex and of all age groups, coming from rural, peri-urban or urban areas, regardless of the form of exposure (vector or oral), with young adults predominating in acute CD cases^10^ ^,^ ^18^. In chronic disease, people of either sex are also diagnosed, but the older age group predominates^12^ ^,^ ^19^. 

## EVOLUTION OF SCIENTIFIC KNOWLEDGE OF THE DISEASE IN AMAZONAS

Over the past two decades, research in Amazonas has expanded knowledge on the transmission, diagnosis and management of CD. The CD and Leishmaniasis Research Group at FMT-HVD, in partnership with UEA and national and international institutions, provides specialized patient follow-up and trains qualified professionals. Studies address the parasite, vectors and educational actions for health promotion. These initiatives are supported financially by Conselho Nacional de Desenvolvimento Científico e Tecnológico (CNPq) and Fundação de Amparo à Pesquisa do Estado do Amazonas (FAPEAM) through the Programa Pesquisa para o SUS (PPSUS) - (FAPEAM/DECIT/MS), CT&I ÁREAS PRIORITÁRIAS (FAPEAM) - UNIVERSAL AMAZONAS (FAPEAM), Priority Areas and Universal programs, strengthening regional healthcare and scientific production.

## AMONG THE MAIN ADVANCES AND DISCOVERIES ACHIEVED ARE:

### Recognition of the genetic diversity and lineages of *Trypanosoma cruzi* in Amazonas

The identification of *T. cruzi* strains in Amazonas has driven research on CD, as this species presents different lineages (Discrete Typing Unit (DTU), from TcI to TcVII)^20^. In the state, TcI and TcIV predominate, with important biological and antigenic differences. TcI is associated with isolated acute and chronic cases, while TcIV predominates in oral transmission outbreaks and circulates solely in humans^10^ ^,^ ^13^ ^,^ ^21^ ^,^ ^22^. Understanding this genetic diversity is essential, as it affects the sensitivity of diagnostic tests, clinical aspects and treatment strategies, highlighting the need for ongoing studies on the parasite.

### Vectors and reservoirs

In Amazonas, the main triatomine species associated with *T. cruzi* transmission is *Rhodnius brethesi*, mainly in the Barcelos region in the upper Negro River^15^ ^,^ ^16^. Other sylvatic species, such as *Panstrongylus geniculatus*, *Eratyrus mucronatus*, *Rhodnius pictipes* and *Rh. robustus*, invade houses near the forests, increasing the risk of transmission of CD. In Manaus, nymphs and adults of *Rh. pictipes* and *Rh. robustus* were found in five palm species, with buriti (*Mauritia flexuosa*) being the most colonized. High natural infection rates with *T. cruzi* TcI have been recorded in these vectors. In addition, the marsupial *Didelphis marsupialis*, common in peri-urban and rural areas, shows high infection rates with the same lineage, reinforcing the close proximity of human populations to the sylvatic cycle of the parasite and the constant risk of exposure to Chagas disease^13^.

### Investigation of oral transmission and active case-finding during outbreaks

Oral transmission of CD in the Amazon, primarily through the consumption of contaminated açaí, has introduced new challenges for epidemiological surveillance, requiring protocols adapted to local conditions. In Amazonas, the low mortality observed in acute cases is associated with early diagnosis performed by the malaria surveillance network. In ten outbreaks, until now, one death has been recorded^10^. However, each outbreak presents specific features that require integrated medical care. Multidisciplinary teams from FMT-HVD and FVS-RCP are involved in patient care, as well as in the epidemiological and entomological investigation of suspected and confirmed cases. A major challenge is post-treatment follow-up: most patients do not receive clinical monitoring due to the lack of specialized outpatient services in their municipalities and the difficulties of traveling to Manaus. As a result, their health status remains undefined, with no adequate monitoring to assess disease progression or potential complications.

In 2022 and 2023, an active search was conducted for patients diagnosed with and exposed to acute Chagas disease outbreaks in five municipalities of Amazonas, locating fewer than 50% of the cases. Blood samples were collected for serological testing and Electrocardiogram (ECG), which detected Immunoglobulin G - (IgG) in patients and exposed individuals, suggesting infection or prior contact with *Trypanosoma cruzi* ^17^. During the visits, educational talks were conducted, along with the presentation of a care flowchart and guidance activities through local media, as well as the training of professionals, contributing to expanding knowledge and strengthening epidemiological surveillance in the region.

The occurrence of CD outbreaks in different municipalities is directly linked to the açaí harvest, highlighting the need for health education programs and good practices in fruit processing^17^. Early diagnosis and integrated surveillance are essential. In Lábrea, in 2018, the laboratory confirmation of viable *T. cruzi* in the açaí that had been consumed reinforced the role of this food as a vehicle of transmission^23^. These findings highlight the importance of strict monitoring and guidance on the preparation and handling of açaí to prevent new cases.

Lack of knowledge about Chagas disease and its modes of transmission is high among vulnerable rural populations and health professionals in Amazonas^18^ ^,^ ^19^. In addition, many health professionals are unaware of the reports of autochthonous CD cases in this region. A recent survey conducted in health services in five municipalities of the metropolitan region of Manaus showed that although 96% of respondents (628 professionals in 71 health units) reported knowing about the disease, 86.6% did not know how to identify its symptoms. In part, this lack of knowledge is due to the absence of records of triatomine colonization inside households.

### Challenges of serological diagnosis of chronic Chagas disease in Amazonas

The diagnosis of CD in the chronic phase is a persistent challenge because it requires serum reactivity in at least two serological tests. In this region, many discrepancies, cross-reactivities and inconclusive results occur. This issue began in 2011 when a serological survey was conducted and it was observed that the conventional serological tests used showed divergent results, limiting the identification of individuals with chronic CD^17^. At the time, variations in the composition of the antigens used were discussed (**Figure 5**).

Smith et al.^18^ showed that the antigens used in serological tests may be inadequate for the TcI and TcIV genotypes predominant in the Amazon, compromising diagnostic sensitivity and specificity. Recent evaluations using commercial kits and in-house methods reinforce these limitations in detecting the chronic phase, highlighting the need to improve regionally tailored serological tools. Ortiz et al.^24^ highlight the potential underdiagnosis of chronic Chagasic cardiomyopathy due to the low sensitivity of conventional methods, reinforcing the urgency of developing more accurate tests for the region.

### Cardiac complications in Chagas disease in Amazonas

Cardiological studies on CD in Amazonas have advanced, expanding the understanding of the pathology^25^ ^-^ ^29^. Since 2009, cases of cardiopathy have been reported, including ventricular aneurysm, systolic dysfunction, cardiorenal syndrome and severe arrhythmia^25^ ^,^ ^29^ as well as records of aborted sudden cardiac arrest^30^. New methodologies, such as tissue Doppler and magnetic resonance imaging, have made it possible to identify subclinical cardiac dysfunctions and the early detection of myocardial lesions even after treatment of the acute phase^31^. The validation of scores such as the Selvester score has contributed to the monitoring of these lesions^32^. Despite these advances, challenges persist, such as the underdiagnosis of Chagas cardiomyopathy, limitations in serological tests and the absence of region-specific policies, which hinders effective disease control even with recent studies on immunogenetics and environmental factors related to its progression^33^.

## BARRIERS AND FACILITATORS FOR THE TREATMENT AND CONTROL OF THE DISEASE

The main obstacles are related to:

### Difficulties in the serological diagnosis of Chagas disease in the chronic phase:

The variety of *T. cruzi* lineages in the region compromises the accuracy of serological tests, generating inconclusive results and possible cross-reactivity, which hinders diagnosis and control, especially in cases of vertical and transfusional transmission.

### Difficulties in the surveillance of vertical transmission “silent disease”:

In Amazonas, there is no screening for CD in women of childbearing age during prenatal care, nor are there data on vertical transmission. Prevalence studies are needed because the absence of surveillance hinders early diagnosis and intervention in cases of maternal-infant transmission.

### Low patient access to post-treatment follow-up services:

In Amazonas, clinical follow-up after acute CD treatment is hindered by transportation barriers, whether by river routes (slow) or air travel (costly and limited), which restricts patient access to services and hampers proper evaluation and effective case management.

### Underreporting and low follow-up for confirmatory testing among blood donors:

The challenges related to diagnostic confirmation among blood donors in Amazonas reflect the difficulties faced by hematology services and the broader health system in achieving accurate and reliable detection of CD. Many individuals who show reactivity during blood donation screening do not seek proper laboratory confirmation, which hampers epidemiological monitoring.

### Structuring and expanding care for Chagas disease in health services:

In this state, there is still no structured and specialized network for the care and follow-up of suspected or confirmed CD cases in the region, except for FMT-HVD, which hinders the continuous monitoring of patients. The absence of a structured and specialized network for care and follow-up represents an obstacle to the continuous monitoring of cases and possible new occurrences, especially in hard-to-reach areas with low capacity for integrated surveillance.

### Low professional awareness of the occurrence of Chagas disease in the region:

There is still a lack of knowledge among the population and especially among health professionals regarding the occurrence of the disease in the region, which may affect early identification and proper management of cases.

### High dissemination and oral transmission:

The rapid transmission of *T. cruzi* through contaminated foods, especially açaí, promotes frequent outbreaks, hindering effective disease control in the region.

### Good practices in the handling and production of palm-based juices, such as açaí:

The production of these juices represents an important source of income, but improved oversight of preparation sites is needed, along with regular training courses on good practices for preparing this type of food.

### Lack of specific health policies and resources:

In Amazonas, there is a shortage of integrated actions and adequate resources for longitudinal, high-quality care, which hinders treatment follow-up and the evaluation of patient outcomes.

### Use of advanced laboratory diagnostics:

Although techniques such as real-time PCR allow the direct detection of *T. cruzi* DNA and are recommended for monitoring parasitemia, evaluating treatment, and determining prognosis in the chronic phase^33^ ^,^ ^34^ -traditionally a laboratory challenge-studies conducted in the state of Amazonas have shown that, to date, this method has not been effective. Low parasitemia is believed to be one of the main limiting factors (Derze, 2025 unpublish data).

Among the main facilitators are:

### Implementation of detection and monitoring actions:

Such as the care flow for CD in the metropolitan region of Manaus, which aims to expand early detection and clinical follow-up, although it is still limited by the impacts of the pandemic.

### Local scientific research and innovation:

Studies involving the assessment of natural infection rates in vectors and reservoirs, investigation of genetic susceptibility factors, and research on oral transmission have the potential to generate knowledge applicable to control and prevention strategies.

## AGENDA FOR PRIORITY RESEARCH ON CHAGAS DESIASES FOR THE COMING YEARS

Research priorities for CD in Amazonas include:

- Improve serological diagnosis, especially in the chronic phase, to make tests more accurate;
- Create a network of specialized outpatient clinics for diagnosis, follow-up and patient management;
- Develop standardized flowcharts for agile and efficient care;
- Implement surveillance of vertical transmission with screening of women of childbearing age, pregnant women and neonates;
- Continuously train health professionals, including through telemedicine, to improve diagnosis and disease control;
- Conduct studies on environmental, social and cultural factors that favor oral transmission, especially through foods such as açaí, in addition to surveying vectors and reservoirs;
- Expand epidemiological and genetic studies of *T. cruzi*;
- Identify the true prevalence of infection in humans, vectors and reservoirs;
- Seek financial support and the opening of public service positions to strengthen human resources and regional epidemiological surveillance.

## FINAL CONSIDERATIONS

Chagas disease remains a concern in Amazonas, with 56% of municipalities already reporting cases, mainly in rural areas. Reducing its incidence requires coordinated, evidence-based efforts to overcome challenges and advance effective disease control.

## Data Availability

Research data is available in the body of the article.

## References

[1] World Health Organization (WHO) (2026). Chagas disease (also known as American trypanosomiasis).

[2] Ministério da Saúde (2025). DATASUS.

[3] Instituto Nacional de Geografia e Estatística (IBGE) (2025). Amazônia Legal.

[4] Barbosa M das GV, Barbosa-Ferreira JMB, Arcanjo ARL, Santana RAG, Magalhães LKC, Mota DT (2015). Chagas disease in the state of Amazonas: History, epidemiological evolution, risks of endemicity and future perspectives. Rev Soc Bras Med Trop.

[5] Ferraroni JJ, Melo JAN de, Camargo ME (1977). Ocorrência de seis casos suspeitos, autóctones sorologicamente positivos. Acta Amazon.

[6] Lescure JP, Emperaire L, Franciscon C (1992). Leopoldinia piassaba Wallace (Arecaceae): A few biological and economic data from the Rio Negro region (Brazil). Forest Ecology and Management.

[7] Rocha DS, Santos CM, Cunha V, Jurberg J, Galvão C (2004). Ciclo biológico em laboratório de Rhodnius brethesi Matta, 1919 (Hemiptera, Reduviidae, Triatominae), potencial vetor silvestre da doença de Chagas na Amazônia [Life cycle of Rhodnius brethesi Matta, 1919 (Hemiptera, Reduviidae, Triatominae), a potential vector of Chagas disease in the Amazon region]. Mem Inst Oswaldo Cruz.

[8] França MS, Frade JM, Konasugawa K, Almeida FB de (1980). Doença de Chagas - primeiro caso autóctone na Amazônia Ocidental - Amazonas - Brasil. Acta Amazon.

[9] Souza-Lima RC, Barbosa MD, Coura JR, Arcanjo AR, Nascimento AS, Ferreira JM (2013). Outbreak of acute Chagas disease associated with oral transmission in the Rio Negro region, Brazilian Amazon. Rev Soc Bras Med Trop.

[10] de Sousa DRT, de Oliveira Guerra JA, Ortiz JV, do Nascimento Couceiro K, da Silva E Silva MRH, Jorge Brandão AR (2023). Acute Chagas disease associated with ingestion of contaminated food in Brazilian western Amazon. Trop Med Int Health.

[11] Monteiro WM, Barbosa MDGV, Guerra JAO, Melo GC, Barbosa LRA, Machado KVA (2020). Driving forces for strengthening the surveillance of Chagas disease in the Brazilian Amazon by "training the eyes" of malaria microscopists. Rev Soc Bras Med Trop.

[12] Magalhães B, Coelho L, Maciel M, Barbosa-Ferreira J, Umezawa E, Coura J (2011). Serological survey for Chagas disease in the rural areas of Manaus, Coari and Tefé in the Western Brazilian Amazon. Rev Soc Bras Med Trop.

[13] Magalhães L, Silveira H, Prestes S, Costa Magalhães LK, Santana RA, Ramasawmy R (2021). Bioecological aspects of triatomines and marsupials as wild Trypanosoma cruzi reservoirs in urban, peri-urban and rural areas in the Western Brazilian Amazon. Med Vet Entomol.

[14] Cucunubá ZM, Gutiérrez-Romero SA, Ramírez JD, Velásquez-Ortiz N, Ceccarelli S, Parra-Henao G (2024). The epidemiology of Chagas disease in the Americas. Lancet Reg Health Am.

[15] Robertson LJ, Havelaar AH, Keddy KH, Devleesschauwer B, Sripa B, Torgerson PR (2024). The importance of estimating the burden of disease from foodborne transmission of Trypanosoma cruzi. PLoS Negl Trop Dis.

[16] Coura JR, Barrett TV, Naranjo MA (1994). Ataque de populações humanas por triatomíneos silvestres no Amazonas: uma nova forma de transmissão da infecção chagásica?. Rev Soc Bras Med Trop.

[17] Coura JR, Peña-Marques MU, Guerra J, Zauza PL, Miguel JC, Pereira JB (2013). A new survey of the serology of human Trypanosoma cruzi infection in the Rio Negro, Brazilian Amazon. A critical analysis. Mem Inst Oswaldo Cruz.

[18] Smith-Doria S, Guerra JA, Guevara-Moctezuma EI (2025). Serological surveillance of orally acquired acute Chagas disease in the Brazilian Amazon using conventional and in house assays. Sci Rep.

[19] Smith-Doria S, Magalhães LKC, Magalhães LKC, Prestes SR, Brandão ARJ, Moura ES (2025). Chronic Chagas Disease in the Brazilian Amazon: Serological Survey, Clinical Follow-Up, and Associated Risk Factors. Am J Trop Med Hyg.

[20] Zingales B, Macedo AM (2023). Fifteen Years after the Definition of Trypanosoma cruzi DTUs: What Have We Learned?. Life.

[21] Monteiro WM, Magalhães LKC, de Sá ARN, Gomes ML, Toledo MJ de O, Borges L (2012). Trypanosoma cruzi IV causing outbreaks of acute chagas disease and infections by different haplotypes in the Western Brazilian Amazonia. PLoS One.

[22] Monteiro WM, Margioto Teston AP, Gruendling AP, dos Reis D, Gomes ML, Marques de Araújo S (2013). Trypanosoma cruzi I and IV Stocks from Brazilian Amazon Are Divergent in Terms of Biological and Medical Properties in Mice. PLoS Negl Trop Dis.

[23] Santana RAG, Guerra MGVB, Sousa DR, Couceiro K, Ortiz J V., Oliveira M (2019). Oral transmission of Trypanosoma cruzi, Brazilian Amazon. Emerg Infect Dis.

[24] Ortiz JV, Couceiro KDN, Doria SS, Sousa DRT, Silveira HMCD, Kesper N (2021). Chagas Cardiomyopathy in the Brazilian Amazon Region: Low Prevalence or Underdiagnosis?. Arq Bras Cardiol.

[25] Ferreira JM, Guerra JA, Md Barbosa (2009). Ventricular aneurysm in a chronic Chagas disease patient from the Brazilian Amazon region. Rev Soc Bras Med Trop.

[26] Ferreira JM, Guerra JA, Magalhães BM, Coelho LI, Maciel MG, Barbosa M (2009). Chronic Chagasic Cardiopathy in Amazon Region: An Etiology to Remember. Arq Bras Cardiol.

[27] Ortiz JV, Pereira BVM, Couceiro KDN, Silva MRH da S, Doria SS, Silva PRL (2018). Cardiac evaluation in the acute phase of chagas’ disease with post-treatment evolution in patients attended in the state of Amazonas, Brazil. Arq Bras Cardiol.

[28] Couceiro KDN, Ortiz JV, Silva MRH da S, Sousa DRT, Andrade RC, Brandão ARJ (2022). Myocardial injury in patients with acute and subacute Chagas disease in the Brazilian Amazon using cardiovascular magnetic resonance. J Am Heart Assoc.

[29] Marques AEAS, Ferreira JMB, Maldonado JA, Santos FGC, Costa DA, Resende GAS (2012). Morte súbita abortada em paciente chagásico crônico da Amazônia Brasileira: relato de caso. Rev Hosp Univ Getúlio Vargas.

[30] Sedlacek EC, Antunes AF, Pereira BVM, Nobre MN, Silva PRL, Silva MRH (2016). Alterations to Tissue Doppler in Patients with Acute Form of Chagas Disease. Arq Bras Cardiol.

[31] Couceiro KN, Ortiz JV, Correia MN, Silva MRHS, Brandão AR, Silva PRL (2021). TheSelvester QRS score as an estimative of myocardial injury in acute chagasic patients from the Brazilian Amazon. BMC Infect Dis.

[32] Guevara Moctezuma EI, Smith Doria S, Ortiz JV, Teixeira de Sousa DR, Mwangi VI, do Nascimento Couceiro K (2023). Chronic Chagas Cardiomyopathy in the Brazilian Amazon region: clinical characteristics and regional distinctiveness. Front Public Health.

[33] Duffy T, Cura CI, Ramirez JC, Abate T, Cayo NM, Nasser JR (2009). Accurate real-time PCR strategy for monitoring bloodstream parasitic loads in Chagas disease patients. PLoS Negl Trop Dis.

[34] Ramírez JD, Duque MC, Guhl F (2013). Trypanosoma cruzi genotypes TcI and TcIV in the Colombian Amazon: evolution of the infection and molecular epidemiology. Trop Med Int Health.

